# ReS_2_ Nanoflowers-Assisted Confined Growth of Gold Nanoparticles for Ultrasensitive and Reliable SERS Sensing

**DOI:** 10.3390/molecules28114288

**Published:** 2023-05-24

**Authors:** Yongping Li, Haohui Liao, Shaobing Wu, Xiaoyu Weng, Yiping Wang, Liwei Liu, Junle Qu, Jun Song, Shuai Ye, Xiantong Yu, Yu Chen

**Affiliations:** State Key Laboratory of Radio Frequency Heterogeneous Integration, College of Physics and Optoelectronic Engineering, Key Laboratory of Optoelectronic Devices and Systems of Ministry of Education and Guangdong Province, Shenzhen University, Shenzhen 518060, China

**Keywords:** ReS_2_ nanoflowers, ReS_2_/AuNPs complexes, surface-enhanced Raman spectroscopy, quantitative detection

## Abstract

ReS_2_, as a new member of transition metal dichalcogenides (TMDCs), has emerged as a promising substrate for semiconductor surface-enhanced Raman spectroscopy (SERS) due to its unique optoelectronic properties. Nevertheless, the sensitivity of the ReS_2_ SERS substrate poses a significant challenge to its widespread application in trace detection. In this work, we present a reliable approach for constructing a novel ReS_2_/AuNPs SERS composite substrate, enabling ultrasensitive detection of trace amounts of organic pesticides. We demonstrate that the porous structures of ReS_2_ nanoflowers can effectively confine the growth of AuNPs. By precisely controlling the size and distribution of AuNPs, numerous efficient and densely packed “hot spots” were created on the surface of ReS_2_ nanoflowers. As a result of the synergistic enhancement of the chemical and electromagnetic mechanisms, the ReS_2_/AuNPs SERS substrate demonstrates high sensitivity, good reproducibility, and superior stability in detecting typical organic dyes such as rhodamine 6G and crystalline violet. The ReS_2_/AuNPs SERS substrate shows an ultralow detection limit of 10^−10^ M and a linear detection of organic pesticide molecules within 10^−6^–10^−10^ M, which is significantly lower than the EU Environmental Protection Agency regulation standards. The strategy of constructing ReS_2_/AuNPs composites would contribute to the development of highly sensitive and reliable SERS sensing platforms for food safety monitoring.

## 1. Introduction

As an ultra-sensitive, non-damaging, and rapid vibrational spectroscopy technology, surface-enhanced Raman spectroscopy (SERS) has a wide variety of applications in food safety, environmental monitoring, and biomedicine [1,2,3,4,5]. Since the initial confirmation by Fleischmann et al. in 1974 that pyridine molecules adsorb onto rough silver surfaces under electrode action [6], SERS technology has undergone extensive research and can now detect a wide range of small-molecule analytes [7,8,9], including proteins, nucleic acids, antibiotics, pesticide residues, and organic pollutants. In comparison to detection methods such as high-performance fluorescence analysis [10], gas chromatography-mass spectrometry (GC-MS) [11], and liquid chromatography (HPLC) [12], SERS exhibits remarkable advantages in terms of rapid and straightforward molecular-specific detection.

Generally, the Raman signal amplification of the target analyte depends on the material properties of SERS substrates. For example, noble metal SERS substrates based on an electromagnetic mechanism (EM) amplify the Raman signal via a unique localized surface plasmon resonance (LSPR) [13,14]. Fu et al. utilized an ultrathin alumina membrane surface patterning technique to fabricate arrays of Ag nanoparticles, achieving an EM-based SERS enhancement factor of up to 10^9^ [15]. Alternately, semiconductor SERS substrates based on a chemical mechanism (CM) amplify Raman signals through charge or energy transfer between the substrate and target molecules [16,17,18]. Muehlethaler et al. demonstrated that the monolayer MoS_2_ exhibits a SERS enhancement factor of 10^5^ through the CM [19]. Therefore, the enhancement of LSPR is typically higher than that of charge transfer by several orders of magnitude [20]; however, the disadvantages of easy agglomeration, instability, and poor biocompatibility have seriously hampered the development of noble metal colloidal SERS substrates.

Recently, 2D semiconductor SERS substrates such as transition metal dichalcogenides (TMDCs) have been extensively studied and developed [21,22,23,24,25,26,27,28]. Thanks to their large surface area, tunable band gap, good biocompatibility, and easy preparation, TMDCs have emerged as ideal SERS substrates for supporting CM. Lv et al. successfully synthesized the monolayer NbS_2_ using the chemical vapor deposition method, demonstrating a superior sensitivity for trace detection compared to graphene [29]. ReS_2_, the new member of the TMDCs, possesses excellent properties that are different from the members of TMDCs such as MoS_2_ owing to its unique 1T’ crystal structure [30,31]. More importantly, ReS_2_ nanosheets maintain direct bandgap semiconductor properties due to the electronic and vibrational decoupling, exhibiting an anisotropic SERS response, and thick-related SERS has been reported in pioneering studies [32,33,34]. Miao et al. proved that the ReS_2_ nanosheets SERS effect is derived from a charge transfer process between ReS_2_ and target molecules. In addition, the SERS sensitivity of ReS_2_ nanosheets decreases with the increase in thickness [32]. Lin et al. confirmed that the angle-dependent Raman enhancement of ReS_2_ with CuPc molecules arises from the anisotropic charge carrier mobility [33]. Meanwhile, Wang et al. reported that the different substrates of ReS_2_ nanosheets can efficiently suppress the fluorescent background of SERS and enable steady detection of the dye molecules at 10^−7^ M [35]. However, although the SERS substrates of ReS_2_ nanosheets exhibit higher homogeneity, better chemical stability, and better biological properties, the Raman enhancement is far less than that of noble metal nanostructures, thus limiting its trace detection capability for target molecules, such as aromatic or toxic compounds.

The construction of novel hybrid or heterostructure SERS platforms combining EM and CM has emerged as an ideal method to obtain higher Raman enhancement [36,37,38,39,40]. For example, Shao et al. fabricated the first ReS_2_ nanocavity-based SERS substrate on gold-modified silicon pyramids by employing a thermal evaporation technique and a hydrothermal method, which showed efficient and stable detection performance in the low-concentration detection of real samples [41]. Furthermore, Liu et al. proved that the 3D nanoflowers structure has a bigger surface area and richer reactive edge sites compared to the nanosheet structure of ReS_2_ [42], rendering it an excellent photocatalyst.

**Figure 1:**
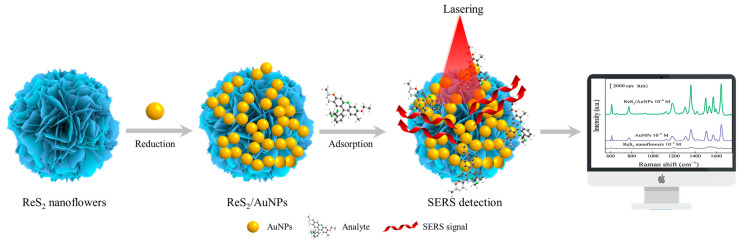
Schematic of preparation and SERS detection of ReS_2_/AuNPs.

Inspired by the above study, a synergistic enhanced SERS substrate of ReS_2_/AuNPs was developed, as shown in Figure 1. ReS_2_ nanoflowers are prepared by hydrothermal synthesis and possess abundant multi-active sites, which exhibit powerful catalytic functions and provide more adsorption sites for the target molecules. In addition, AuNPs can grow uniformly on the surface of ReS_2_ nanoflowers without reducing agents and bind to them through Au–S covalent bonds. The high sensitivity of SERS detection was achieved by controlling the size and gap of the AuNPs and then determining the specific enhancement mechanism of the SERS effect. The two representative organic dye molecules of crystalline violet (CV) and rhodamine 6G (R6G) were selected for evaluation of the analytical ability and SERS performance on the ReS_2_/AuNPs substrate, and applied to organic pesticide detection. The results showed that the detection limits of the ReS_2_/AuNPs composites for R6G, CV, and tetramethylthiuram disulfide (TTD) were as low as 10^−10^ M, with detection deviations ranging from 14.7% to 15.3%. This demonstrates that the ReS_2_/AuNPs complexes have excellent sensitivity, reproducibility, and stability, making it feasible for trace determination of organic pollutants with high sensitivity and stability.

## 2. Results and Discussion

### 2.1. Preparation Process and Characterization Analysis of ReS_2_/AuNPs Complexes

**Figure 2:**
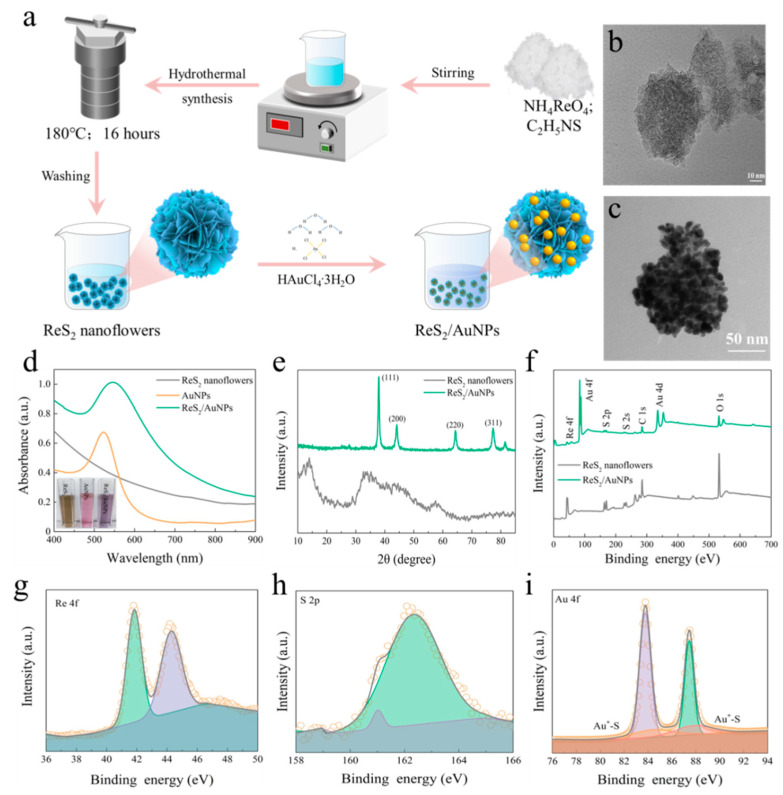
(**a**) Preparation process of ReS_2_/AuNPs composite substrate. TEM images of (**b**) ReS_2_ nanoflowers, and (**c**) ReS_2_/AuNPs reduced at 0.08 mM HAuCl_4_. (**d**) Absorption spectra of ReS_2_ nanoflowers, AuNPs, and ReS_2_/AuNPs, reduced at 0.28 mM HAuCl_4_. (**e**) XRD spectra of ReS_2_ nanoflowers and ReS_2_/AuNPs. (**f**) XPS survey spectra for ReS_2_ nanoflowers and ReS_2_/AuNPs, (**g**) Re 4f, (**h**) S 2p, and (**i**) Au 4f in ReS_2_/AuNPs.

Figure 2a shows the preparation process for the ReS_2_/AuNPs complexes. In order to uniformly attach the appropriate amount of AuNPs to the ReS_2_ nanoflowers without using a reducing agent, a large specific surface area and strongly catalytic properties of ReS_2_ nanoflowers were prepared via a hydrothermal synthesis at a temperature of 180 °C for 16 h. Then, under constant temperature at 80 °C, upon the addition of a certain concentration of HAuCl_4_ solutions, the color of the mixed solution gradually changed from pale yellow to deep purple (as illustrated in Figure 2d), indicating the successful reduction of AuNPs and synthesis of the ReS_2_/AuNPs complexes. Figure 2b,c display the transmission electron microscopy (TEM) images of the ReS_2_ nanoflowers and ReS_2_/AuNPs composites at appropriate magnification voltages ranging from 40 to 120 kV. The results show that the flower-like ReS_2_ has an average particle size of 49.5 nm, which uniformly adhered in parallel without stacking and dispersed homogeneously, as shown in Figure S1. The three-dimensional surface area of the ReS_2_ nanoflowers provides a number of adsorption sites for AuNPs, resulting in the reduced AuNPs being grown uniformly on its surface (shown in Figure 2c).

In addition, Figure 2d shows the UV–Vis absorption images of the ReS_2_ nanoflowers, AuNPs, and ReS_2_/AuNPs complexes at 400–900 nm. The results indicate that no distinctive characteristic peaks were found in the absorption spectra of ReS_2_ nanoflowers; however, a weak small shoulder peak appeared at 738 nm (Figure 2d). Compared to the ReS_2_ nanoflowers and AuNPs, the overall absorption intensity of the ReS_2_/AuNPs increased with a red-shift of the longitudinal LSPR peak to 545 nm with lateral broadening, indicating that the AuNPs of ReS_2_/AuNPs clustered on the surface of ReS_2_ nanoflowers. The small shoulder peak at 738 nm disappeared, indicating the successful complexation of the ReS_2_ nanoflowers with AuNPs. Figure 2e presents the X-ray diffraction (XRD) spectra of the ReS_2_ nanoflowers and the ReS_2_/AuNPs powder. Four diffraction peaks at 2*θ* = 13.9°, 34.9°, 44.5°, and 57.5° correspond to the (100), (002), (300), and (−122) crystallographic planes of ReS_2_, respectively, indicating the successful synthesis of ReS_2_ nanoflowers [43]. Following the involvement of AuNPs, four peaks were observed at 38°, 44.2°, 64.4°, and 77.6°, which are located in the (111), (200), (220), and (311) crystal planes of planar cubic Au [44].

Moreover, XPS spectra showed typical Re 4f, Au 4f, and S 2p characteristic diffraction peaks derived from the ReS_2_/AuNPs, as shown in Figure 2f,i. In contrast to the S 2p of ReS_2_ nanoflowers (Figure S2), the S 2p_3/2_ and S 2p_1/2_ peaks of ReS_2_/AuNPs were blue-shifted to 161 and 162.3 eV, respectively, which indicates the important synergistic role of the ReS_2_ nanoflowers in Au reduction. Furthermore, Figure 2i shows the bond energy peaks of Au 4f located at 83.7 and 87.4 eV with a binding energy difference of 3.7 eV, suggesting the successful Au reduction, whereas the symmetric peaks at 84.4 and 87.8 eV indicate the AuNPs adsorption with the ReS_2_ nanoflowers via Au–S bonds. As the most direct evidence, EDS images of Re, S, and Au elements confirm the formation of the ReS_2_/AuNPs complexes (Figure S1), and the elemental ratio values for Re, S, and Au were 1.5, 2.5, and 96%, respectively.

### 2.2. The Controlled Growth of AuNPs Assisted by ReS_2_ Nanoflowers

**Figure 3:**
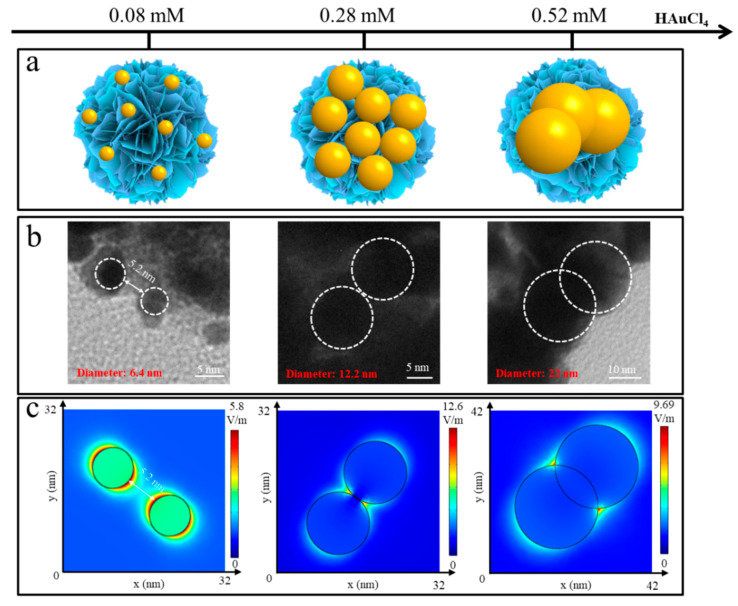
(**a**) Diagram of ReS_2_/AuNPs with different AuNPs sizes. (**b**) TEM images of AuNPs with varying sizes and gaps. (**c**) Electric field intensity distribution corresponding to (**b**). Gradient color bar indicates the electric field intensity of E/E_0_.

Figure 3b and Figure S3 display the TEM images of ReS_2_/AuNPs with different sizes of AuNPs. Under continuous stirring at a constant temperature of 80 °C, controlled ReS_2_/AuNPs composites with average sizes of 6.4 nm, 12.2 nm, and 22 nm were obtained by adding HAuCl_4_ solutions with concentrations of 0.08 mM, 0.28 mM, and 0.52 mM, respectively, to a 5 mL ReS_2_ nanoflowers solutions. In addition, the UV–Vis absorption spectra of ReS_2_/AuNPs with different sizes of AuNPs indicated that as the AuNPs’ size enlarges, the LSPR peak is red-shifted from 524 nm to 563 nm. In addition, various particle sizes of AuNPs show the different particle gaps on the surface of ReS_2_ nanoflowers, which is essential for the generation of “hot spots” with different intensities. Typically, the higher the concentration of HAuCl_4_, the larger the size of AuNPs aggregated on ReS_2_ nanoflowers. However, with the increasing HAuCl_4_ concentrations, the limited surface area of the ReS_2_ nanoflowers was unable to adsorb numerous large-sized AuNPs, resulting in the aggregation and overlapping of AuNPs, which may cause the intensity of the “hot spot” to decrease.

Subsequently, the plasma oscillation process of the 514 nm laser excitation was simulated using finite elements to analyze the electromagnetic field intensity (E) distribution for different AuNPs sizes and gaps. The simulation conditions were as follows: three-dimensional frequency-domain modeling was utilized with the incident light vertically propagating along the Z-axis, initially polarized along the X-axis, and the light intensity set to 1 V/m. The solution domain was a cube with dimensions of 800 nm × 800 nm × 800 nm. Perfectly conducting boundaries were employed to eliminate scattering waves in all boundary directions. Figure 3c shows that, when the AuNP’s particle gap was larger than 5.2 nm, the E value was lower than 5.8 V/m, which is possibly related to weak LSPR. Moreover, the highest E value was 12.6 V/m, with gaps close to zero, which is attributed to the strong Au–S bonding interactions that formed tight and high-efficient “hot spots” for the 12.2 nm AuNPs on the surface of the ReS_2_ nanoflowers. Following the overlap of AuNPs, the E value gradually decreased and did not enhance the E value further, presumably owing to the dipole oscillations preventing the formation of a highly effective “hot spot” between the two contacting AuNPs [45].

### 2.3. Feasibility and Sensitivity Analysis of the ReS_2_/AuNPs SERS Substrate

**Figure 4:**
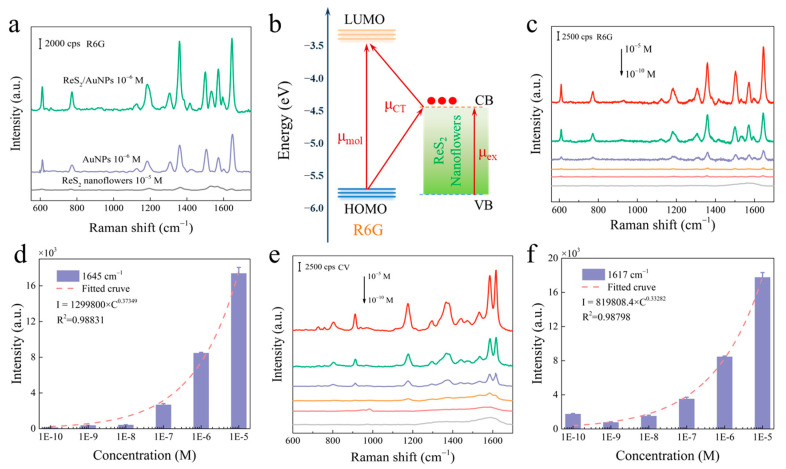
(**a**) The R6G SERS spectra of the ReS_2_ nanoflowers, AuNPs, and ReS_2_/AuNPs substrate detection. (**b**) Schematic of charge transfer from the ReS_2_ nanoflowers to R6G. Concentration-dependent SERS spectra based on (**c**,**d**) R6G and (**e**,**f**) CV Raman characteristic peaks and their intensity-concentration relationships.

Under optimized conditions, the enhanced sensing performance of ReS_2_/AuNPs as a SERS substrate was verified by detecting R6G and CV dye molecules. First, 10^−5^, 10^−6^, and 10^−6^ M concentrations of R6G solutions were soaked with ReS_2_ nanoflowers, AuNPs, and ReS_2/_AuNPs, respectively, for 1 h. After natural drying, SERS measurements were performed under the 514 nm excitation light. Figure 4a shows that the R6G Raman characteristic peaks at 611, 773, 1360, 1510, and 1645 cm^−1^ were detected for the ReS_2_ nanoflowers, AuNPs, and ReS_2_/AuNPs SERS substrates [46]. Notably, the ReS_2_ nanoflowers as a semiconductor SERS substrate could detect capable 10^−5^ M R6G, which is attributed to the CM of the ReS_2_ nanoflowers and the abundance of active sites. According to a previous study, the conduction band minimum (CBM) and valence band maximum (VBM) of the bilayer ReS_2_ were −4.46 and −5.86 eV, respectively [47]. The highest occupied molecular orbital (HOMO) energy levels and the lowest unoccupied molecular orbital (LUMO) energy levels of R6G were −5.7 and −3.4 eV, respectively [48]. When the excitation light irradiated the ReS_2_ nanoflowers surface, the electrons of VBM jumped to the CBM [49] and were rapidly transferred to the LUMO of the R6G dye molecules (Figure 4b), which makes the R6G dye molecules obtain a CM order-of-magnitude SERS enhancement.

When the EM of AuNPs participated, the ReS_2_/AuNPs substrate formed an effective “hot spot,” which enhanced the Raman signal by two times compared to that of AuNPs (Figure S4). In addition, the Raman signal of 10^−10^ M R6G was detected by ReS_2_/AuNPs (Figure 4c) with the synergistic interaction of the local EM field generated by AuNPs and rapid charge transfer of ReS_2_/AuNPs complexes. Figure 4c,e show the variation in the SERS sensing ability of ReS_2_/AuNPs substrate with different R6G and CV concentrations (10^−6^–10^−10^ M). The SERS intensities of R6G and CV were reduced with reducing concentration, and the SERS signals from the strongest characteristic peaks of R6G and CV could still be measured at concentrations lower than 10^−10^ M. In addition, the Raman characteristic peaks of R6G at 1645 cm^−1^ and CV at 1617 cm^−1^ were selected for fitting the Lorentz function to investigate the dependence between SERS intensity and concentration. Figure 4d,f show that the SERS intensity obeyed a power law relationship with fitted correlation coefficient (R^2^) values greater than 0.9; the fitting formulas were I_R6G_ = 1299800 × C^0.37349^, I_CV_ = 819808.4 × C^0.33282^. These results indicate that the ReS_2_/AuNPs substrate has ultrahigh detection sensitivity for target molecules in the 10^−6^–10^−10^ M range.

### 2.4. Stability Analysis of the ReS_2_/AuNPs SERS Substrate

**Figure 5:**
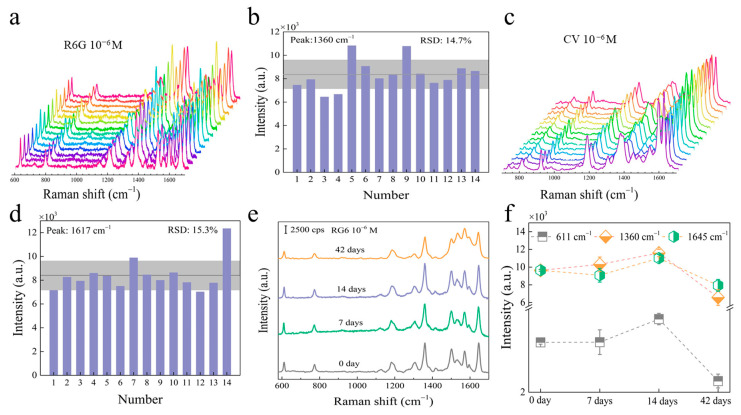
SERS spectra of (**a**) R6G and (**c**) CV from ReS_2_/AuNPs substrate, and corresponding histograms for the characteristic peak intensity at (**b**) R6G and (**d**) CV. Note that in (**a**,**c**), the different colored curves represent the SERS spectra obtained from different sites tested. (**e**) SERS spectra of R6G at different storage times, and (**f**) characteristic peak intensities at different storage times.

An ideal SERS sensor requires high sensitivity, as well as great repeatability and stability of the substrate. Therefore, to evaluate the repeatability of ReS_2_/AuNPs complexes, 14 detection sites were randomly selected from the substrate with adsorbed 10^−6^ M R6G and CV, as shown in Figure 5a,c. The results show that the SERS spectra of both R6G and CV exhibit consistent Raman characteristic peaks similar to those shown in Figure 4. In addition, the Raman signal intensities were relatively consistent. Figure 5b,d present the relative standard deviation (RSD) of the Raman peaks at 1360 cm^−1^ and 1617 cm^−1^ for R6G and CV in Figure 5a,c, respectively. The results demonstrated that the RSD of R6G and CV was 14.7% and 15.3%, respectively, suggesting great repeatability on the ReS_2_/AuNPs substrate. In addition, Figure 5e displays the SERS spectra obtained by testing the ReS_2_/AuNPs substrate adsorbed with R6G dye molecules, which was stored in the dark at room temperature, with measurements taken at seven-day intervals. The results show that the Raman signal intensity of RG6 did not significantly change even after the substrate was stored for more than a month, and the position of the peaks is not shifted in any way. Figure 5f demonstrates that the peak intensities of 6RG at 611 cm^−1^, 1360 cm^−1^, and 1645 cm^−1^ did not show any significant decrease after 14 days. Even after 42 days, only a minimal decline was observed. This indicates the high stability of the ReS_2_/AuNPs SERS substrate.

### 2.5. The Detector Range and Practical Applications of ReS_2_/AuNPs SERS Substrate

**Figure 6:**
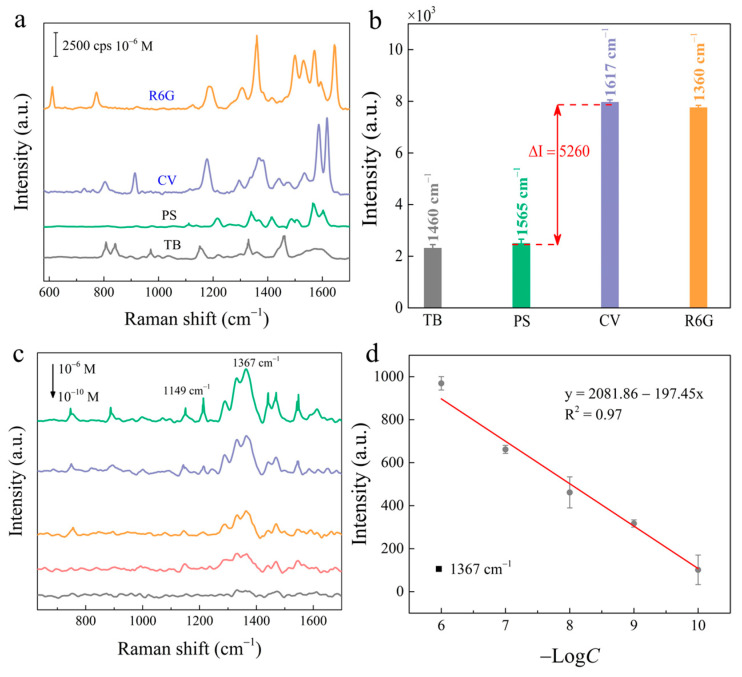
(**a**) SERS spectra of ReS_2_/AuNPs substrate detecting 10^−6^ M cationic dyes (R6G, CV) and anionic dyes (PS, TB). (**b**) The intensity histograms of the Raman characteristic peaks for cations and anions. (**c**) SERS spectra of different concentrations of TTD solutions from the ReS_2_/AuNPs substrate detection. (**d**) Relationship between concentrations of TTD solutions and SERS signal intensity.

First, anions and cationic dyes were utilized to study and analyze the suitability of the ReS_2_/AuNPs substrate for different kinds of surface charge target molecules. Figure 6a shows the SERS spectra of the ReS_2_/AuNPs solution immersed in a mixture of 10^−6^ M R6G, CV, ponceau S (PS), and trypan blue (TB) dye molecules after one hour. Figure 6b represents the SERS intensities of TB, PS, CV, and R6G at characteristic peaks located at 1460 cm^−1^, 1565 cm^−1^, 1617 cm^−1^, and 1360 cm^−1^, respectively. The results indicate that, although the ReS_2_/AuNPs SERS sensor was reduced in sensitivity to anions (PS, TB) compared to cationic dyes (R6G, CV), very complete SERS spectra of anionic dyes were still detected. In addition, the well-known Raman characteristic peaks of PS and TB appear at 1565 cm^−1^ and 1460 cm^−1^, respectively. This result indicates that the ReS_2_/AuNPs complexes are suitable for SERS detection of analytes with different surface charges, without the assistance of ligands.

The feasibility of the ReS_2_/AuNPs SERS sensor for food safety applications was further investigated using TTD, a common organic pesticide. TTD is frequently used as a protective agent against normal crop growth; however, excessive residues in crops cause neurological damage in humans. The TTD molecules were immersed within the ReS_2_/AuNPs substrate for 1 h, dried naturally, and subjected to SERS detection. Figure 6c shows the SERS spectra of various concentrations of TTD (10^−6^–10^−10^ M), which exhibited two distinctive Raman characteristic peaks. The peak located at 1367 cm^–1^ is associated with C–N stretching and CH_3_ symmetric deformation vibration modes [50]. The intensity of the SERS signal decreased with decreasing concentrations of TTD, and the characteristic Raman peak at 1367 cm^−1^ was still observed at 10^−10^ M. For further quantitative analysis, Figure 6d shows the dependence of the SERS intensity at 1367 cm^−1^ on the TTD concentrations. The SERS intensity is linearly related to the negative logarithm of concentration with R^2^ of 0.97, and the fitted equation is I = 2081.86 + 197.45 Log *C*. The detection limit of 10^−10^ M is significantly lower than the residue limit of 3 ppm for fruits and vegetables set by the EU Environmental Protection Agency [51].

## 3. Materials and Methods

### 3.1. Materials

Thioacetamide (CH_3_CSNH_2_, 99.09%), CV, and R6G were bought from Aladdin Bio-Chem Technology Co., Ltd. (Shanghai, China). Ammonium perrhenate (NH_4_ReO_4_, 99.99%) was obtained from Weng Jiang Reagent Company (Shaoguan, China). HAuCl_4_·3H_2_O and TTD (97.5%) were purchased from Macklin (Shanghai, China). The carboxymethylcellulose sodium (CMC-Na, 99.5%), PS, and trypan blue TB were obtained from Dow Corporation (Midland, MI, USA), Shanghai Yuanye Bio-Technology Co., Ltd. (Shanghai, China), and Beyotime Biotechnology (Shanghai, China), respectively. Aqueous solutions of 15 nm gold nanoparticles were purchased from Hangzhou Xinqiao Biotechnology Co., Ltd. (Hangzhou, China).

### 3.2. Synthesis of Three-Dimensional ReS_2_ Nanoflowers

ReS_2_ nanoflowers were prepared using a hydrothermal synthesis method [43]. Briefly, 134.12 mg of NH_4_ReO_4_ powder was mixed with 35 mL of deionized water (18.2 MΩ) and stirred continuously at room temperature. For 10 min, 150.26 mg of CH_3_CSNH_2_ was added to the above solution. After continuous stirring for 15 min, the mixed solution was poured into a polytetrafluoroethylene liner contained in a stainless steel autoclave and the reaction was continued for 16 h at 180 °C. With the temperature of the reaction chamber dropped to 25 °C, the reactants were washed by centrifugation with anhydrous ethanol and deionized water at 11,000 rpm 4 times. The collected sediment was dried in a thermostatic drying oven at 60 °C for 24 h to obtain ReS_2_ nanoflowers in powder form. Subsequently, 2.5 mg of the ReS_2_ nanoflowers powder was dispersed in 100 mL of deionized water, resulting in a ReS_2_ nanoflowers solution with a concentration of 0.025 mg/mL.

### 3.3. Synthesis of the ReS_2_/AuNPs Complexes

First, 500 μL of 50 mM CMC-Na was added into 5 mL of 0.025 mg/mL ReS_2_ nanoflowers solution and continuously stirred at room temperature for 5 min. Subsequently, 40, 140, and 260 μL (0.08, 0.28, 0.52 mM) of HAuCl_4_ were placed into the solution and continuously stirred for 10 min at 80 °C. When the solution undergoes a color change (light red, deep purple, or dark blue), it indicates the successful reduction of AuNPs. Then, the excess CMC-Na was removed by centrifugal washing with deionized water at 13,000 rpm three times. Finally, the ReS_2_/AuNPs complexes were dried at 60 °C for 12 h.

### 3.4. Materials Characterization

The scanning electron microscope (SEM), energy spectrum (EDS), and TEM images of ReS_2_/AuNPs were examined using scanning electron microscopy (SEM, Supra 55 Sapphire, Oberkochen, Germany) operated at 15 kV, and transmission electron microscopy (TEM, HITECH HT7700, Tokyo, Japan) operated at 40 kV. The elemental composition and crystal phase of ReS_2_/AuNPs were obtained from X-ray photoelectron spectroscopy (XPS, Thermo Scientific NEXSA, Waltham, MA, USA) and high-resolution X-ray diffractometer (XRD, X′pertpro, Amsterdam, The Netherlands). The absorption spectra of ReS_2_/AuNPs were measured with a UV-1780 spectrometer.

### 3.5. SERS Experiments

First, 100 μL of solutions with different concentrations of R6G, CV, TB, and PS were mixed with 0.25 mg of ReS_2_/AuNPs powder. After sufficient adsorption for 1 h, ReS_2_/AuNPs powder was cleaned twice with deionized water for the removal of unabsorbed dye molecules. Subsequently, the solutions were placed on a slide and allowed to dry. A Renishaw inVia confocal Raman spectrometer was employed for SERS detection. Finally, the conditions for Raman testing were as follows: operating wavelength 514 nm; laser power 10%; 50× objective lens; integration time 20 s; an acquisition range of 500–2000 cm^−1^. Furthermore, taking TTD as an example, the practical application performance of ReS_2_/AuNPs in SERS was evaluated. The ReS_2_/AuNPs substrate was immersed in TTD solutions with varying concentrations for one hour at room temperature, followed by cleaning and air drying before testing. The electric field distribution of ReS_2_/AuNPs was simulated by the finite-element method.

## 4. Conclusions

In conclusion, a three-dimensional flower-like ReS_2_ nanostructure with an abundance of active sites was successfully synthesized on a large scale using a hydrothermal method. Assisted and controlled by the ReS_2_ nanoflowers, AuNPs were uniformly, densely, and zero-spaced anchored on the surface of the ReS_2_ nanoflowers; therefore, a ReS_2_/AuNPs SERS platform with a considerable number of “hot spots” was formed. The results show that the SERS enhancement of the ReS_2_/AuNPs substrate originated from the synergistic enhancement of EM and CM, which indicated excellent sensitivity, good reproducibility, and excellent storage stability for the representative organic dyes R6G and CV. In practical applications, the ReS_2_/AuNPs substrate can linearly detect organic pesticide molecules, (such as TTD) at concentrations as low as 10^−10^ M, exhibiting promising applications in food safety and environmental detection.

## Data Availability

Not applicable.

## Supplementary Materials

The following supporting information can be downloaded at: https://www.mdpi.com/article/10.3390/molecules28114288/s1, Figure S1: (a–b) TEM and (c) SEM images of ReS_2_ nanoflowers. (d) SEM images and (e–f) EDS images of ReS_2_/AuNPs complexes; Figure S2: XPS spectra for ReS_2_ nanoflowers—(a) Re 4f, (b) S 2p; Figure S3: TEM images, average particle size, and absorption spectra of ReS_2_/AuNPs complexes with different concentrations of HAuCl_4_; Figure S4: (a–b) Histogram of the intensity of the R6G characteristic peak corresponding to Figure 4a.

## Sample Availability

Samples of the ReS_2_ nanoflowers and ReS_2_/AuNPs complexes are available from the authors.
