# Supplementary material for: ReS2 Nanoflowers-Assisted Confined Growth of Gold Nanoparticles for Ultrasensitive and Reliable SERS Sensing
Source: Molecules. 2023 May 24;28(11):4288. doi: 10.3390/molecules28114288 (PMC10254201; doi:10.3390/molecules28114288)
Supplement: Supplementary file 1 [file molecules-28-04288-s001.zip › molecules-2390928-supplementary.pdf]

ReS_2 Nanoflowers-Assisted Confined Growth of Gold Nanoparticles for Ultrasensitive and Reliable SERS Sensing

Yongping Li †, Haohui Liao †, Shaobing Wu, Xiaoyu Weng, Yiping Wang, Liwei Liu, Junle Qu, Jun Song, Shuai Ye *, Xiantong Yu * and Yu Chen *

State Key Laboratory of Radio Frequency Heterogeneous Integration, College of Physics and Optoelectronic
Engineering, Key Laboratory of Optoelectronic Devices and Systems of Ministry of Education and Guangdong
Province, Shenzhen University, Shenzhen 518060, China; liyongping2020@email.szu.edu.cn (Y.L.); liaohaohui2022@email.szu.edu.cn (H.L.); wshaobing92@163.com (S.W.); xiaoyu@szu.edu.cn (X.W.); ypwang@szu.edu.cn (Y.W.); liulw@szu.edu.cn (L.L.); jlqu@szu.edu.cn (J.Q.); songjun@szu.edu.cn (J.S.)

* Correspondence: yes121@szu.edu.cn (S.Y.); xtyu@szu.edu.cn (X.Y.); chenyu2016@szu.edu.cn (Y.C.)

† These authors contributed equally to this work.

1. Supplementary Figures

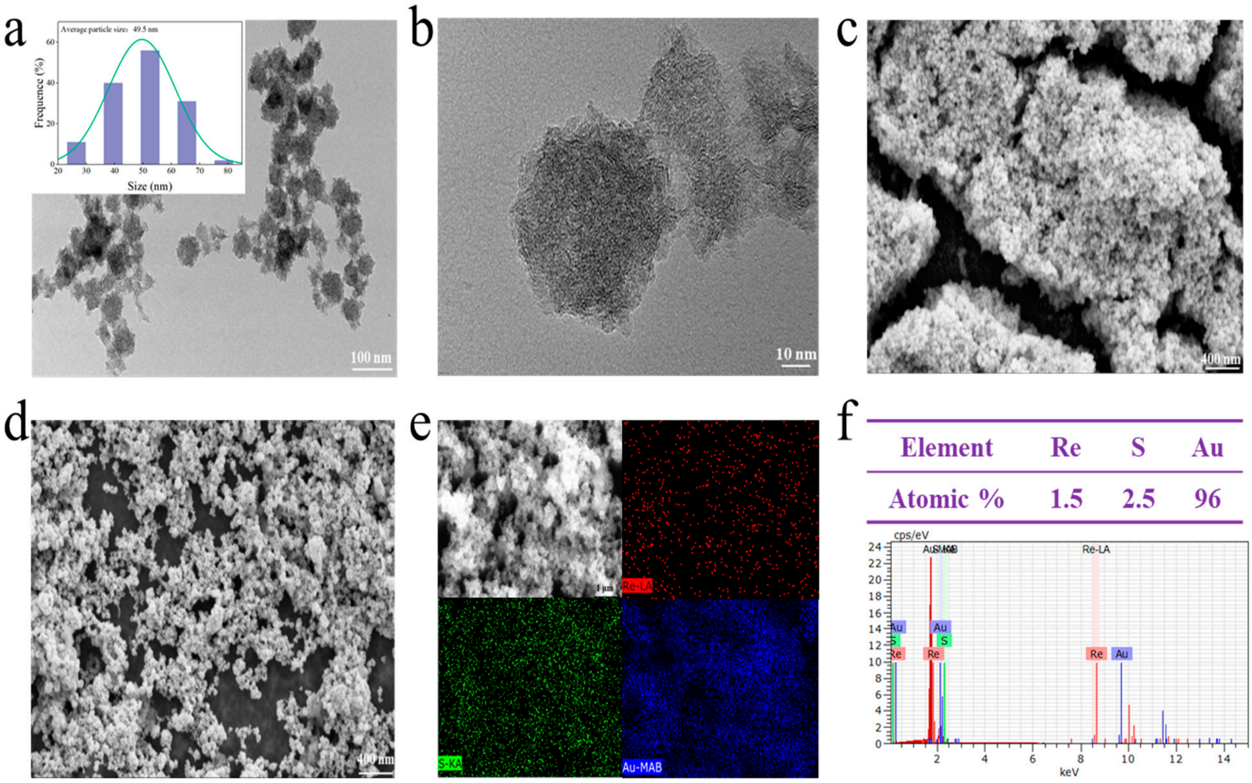

Figure S1. a-b TEM and **c** SEM images of ReS_2 namoflowers. **d** SEM images and **e-f** EDS images of ReS_2/AuNPs complexes.

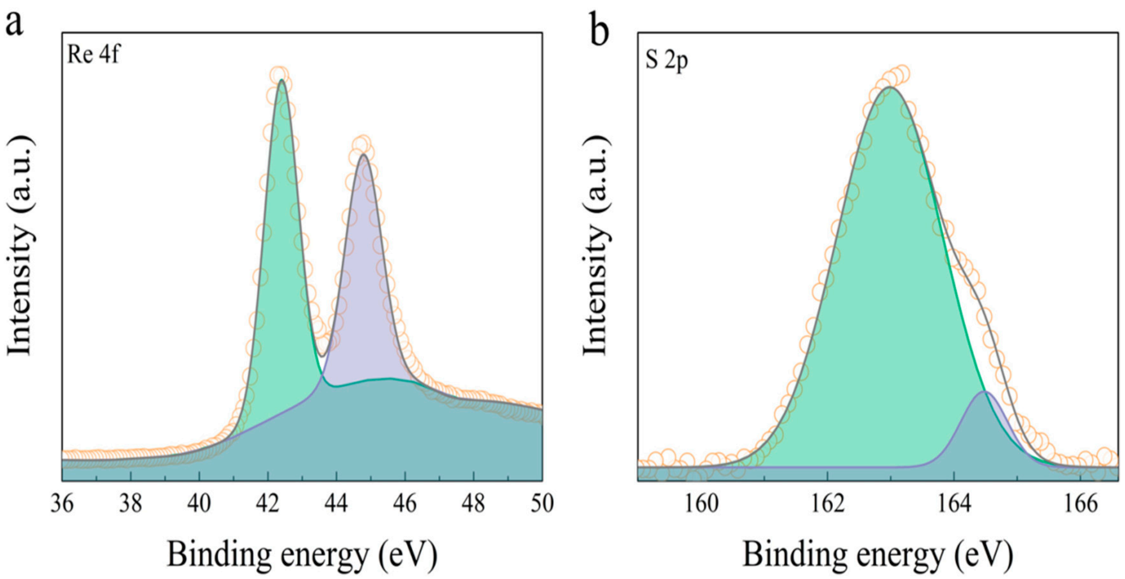

Figure S2. XPS spectra for ReS_2 nanoflowers: **a** Re 4f, **b** S 2p.

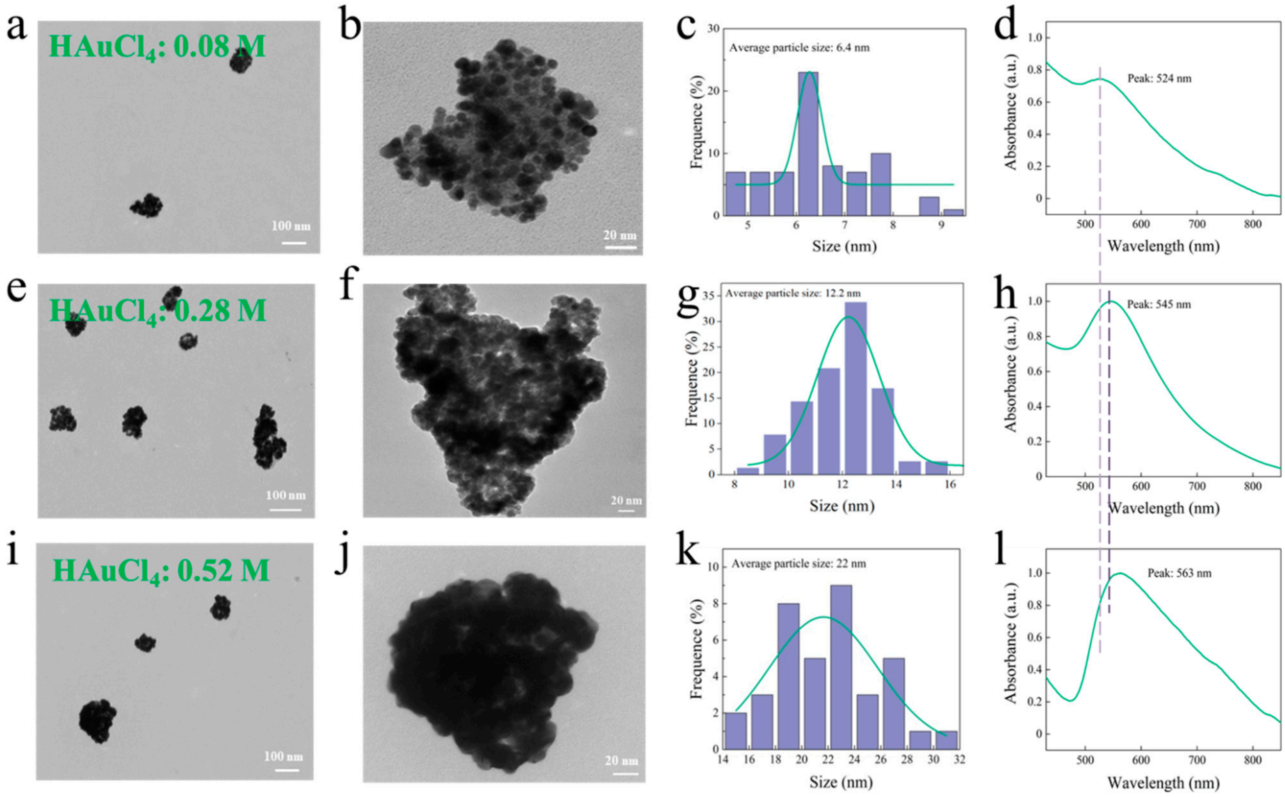

Figure S3. TEM images, average particle size, and absorption spectra of ReS_2/AuNPs complexes with different concentrations of $HAuCl_4$.

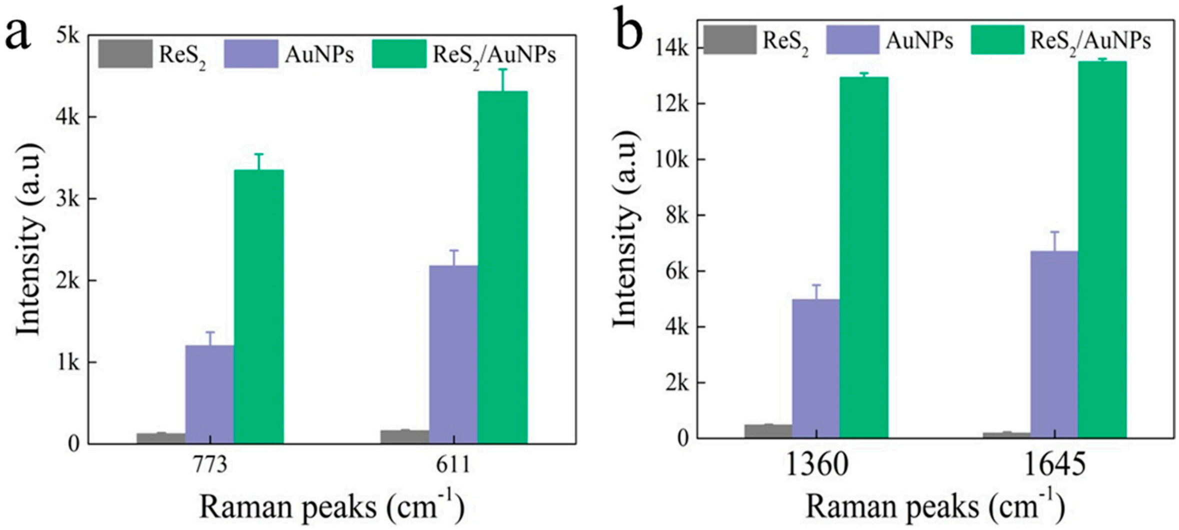

Figure S4. a-b Histogram of the intensity of the R6G characteristic peak corresponding to Figure 4a.

2. Supplementary Formula

Relative standard deviation (RSD) calculation formula.

$$RSD = SD/I_p \tag{S-1}$$

The SD is the standard deviation of the intensity, and I_p is the average SERS intensity of the characteristic peak.
